# Classifying the Integration of Healthcare Providers and Insurers

**DOI:** 10.1002/hec.70019

**Published:** 2025-07-22

**Authors:** Xiaodan Liang, John Mullahy

**Affiliations:** ^1^ Department of Population Health Sciences University of Wisconsin School of Medicine and Public Health Madison Wisconsin USA

**Keywords:** healthcare, insurer, provider, vertical integration

## Abstract

The value‐based payment reform prompted by the Affordable Care Act has stimulated vertical integrations of healthcare providers and insurers. The consequences of these integrations may vary significantly depending on the markets and entities involved. This article points out the limitations of familiar binary classifications for provider‐insurer integrations in prior studies. To address these limitations, we propose a framework and taxonomy that include four key aspects for examining variations in provider‐insurer integrations. The first is from the care delivery perspective; it sheds light on levels of care services owned by an integrated system and their variation across regions within the system. The second is from the insurance markets' perspective; it pertains to insurance markets in which an integrated system competes. The third is from the organizational perspective; it points out that whether the insurer or the provider is dominant in an integrated system may affect the system's priorities—care delivery reform or cost containment. The last highlights the dynamics of integrated systems that can involve the other three. We offer these insights and their possible applications hoping to sharpen discussion and research on provider‐insurer integrations, and to assist antitrust agencies in evaluating relevant legal cases under the 2023 Merger Guidelines.

## 1 Introduction

Vertical integrations (VIs) of healthcare providers and insurers in the U.S. have existed as early as the nineteenth century (Supporting Information S1: Appendix Section 1). Over the years, provider‐insurer integrations have experienced several waves, with the latest driven by the paying‐for‐value under the Affordable Care Act (ACA) (Allan Baumgarten, LLC 2017). By 2021, one‐third of U.S. health systems were offering insurance products (AHRQ 2024). VIs of healthcare providers and insurers have arisen in other countries, such as Chile and Greece (Noton and Stenimachitis 2022). However, this paper focuses exclusively on the U.S.

Provider‐insurer integrations could mitigate contractual problems, align incentives, and facilitate care coordination between the integrated provider and insurer, all of which can improve care quality and reduce unnecessary care use (Williamson 1971; Holmstrom 1999; Hart and Holmstrom 2010; Eggleston et al. 2004; Enthoven and Baker 2018; Agha et al. 2023). However, provider‐insurer integrations are not exempt from antitrust concerns (Douven et al. 2011; Cuesta et al. 2024; De Stefano and Salinger 2024).

Economic models indicate that VIs' impacts vary significantly by market and entities (De Stefano and Salinger 2024; Perry 1989; Bresnahan and Levin 2013), highlighting the importance of considering variations in provider‐insurer integrations. Prior empirical studies have mostly used a binary classification (Frakt et al. 2013; Howard et al. 2018; La Forgia et al. 2018; Meyers et al. 2020), which simplifies analysis but may obscure important variation.

This paper proposes a framework and taxonomy for examining variations in provider‐insurer integrations, with the hope of sharpening discussions and empirical research. It considers four aspects: care delivery, insurance markets, organizational management, and the dynamics of integrated systems.

In this article, the *provider* refers to a healthcare organization, for example, a health system, hospital, or physician group. Either a *provider‐insurer integrated system* or an *integrated system* refers to a system formed through provider‐insurer integration. An *integrated* provider (or insurer) is one that belongs to such a system.

## 2 Economic Models of Provider‐Insurer Integration: A Very Brief Overview

Economists model VI in both organizational economics and industrial organization research. Organizational economics views VI as an efficient response to contractual issues (Bresnahan and Levin 2013). Coase (1937) argued that firms' sizes depend on the extent to which they offer transaction cost advantages. Williamson (1971) claimed that market contracts cannot cover all contingencies, leading to conflicts between separate entities. VI allows these conflicts to be resolved internally. Additionally, VI enables firms to restructure the incentives of previously separated units (Holmstrom 1999), and facilitates information transfer and coordination (Hart and Holmstrom 2010; Williamson 1971).

Industrial organization research suggests that VI is jointly determined by the firm and the horizontal structure of the constituent industries (Bresnahan and Levin 2013). VI can be driven by benefits from scale economies (Bresnahan and Levin 2013; Levy 1984). Firms may adopt VI to address market imperfections, such as eliminating double marginalization (Spengler 1950), or internalizing externalities (Perry 1989; Mathewson and Winter 1984). Although VI could increase welfare, it is not exempt from anti‐competitive concerns, such as raising rivals' costs and creating barriers to market entry (Bresnahan and Levin 2013; De Stefano and Salinger 2024; Perry 1989).

For provider‐insurer integrations, economists have modeled the effects of insurer competition on efficiency gains from the integration (Eggleston et al. 2004). They have also developed models focusing on anti‐competitive concerns, addressing the incentives and effects of exclusive contracting and integration (Cuesta et al. 2024; Douven et al. 2011).

These economic models suggest provider‐insurer integrations can either increase or decrease welfare, depending on product characteristics, market features, and customer traits. As such it is informative to consider variations in VIs of providers and insurers when assessing their effects.

## 3 Definitions of Provider‐Insurer Integration

Perry (1989) distinguished between *full* and *partial* VI, with the former emphasizing the exclusivity of the intermediate product. Since only a few provider‐insurer integrations can afford an exclusive relationship (Allan Baumgarten, LLC 2017), this article uses *VI* or *integration* to refer to both.

**FIGURE 1:**
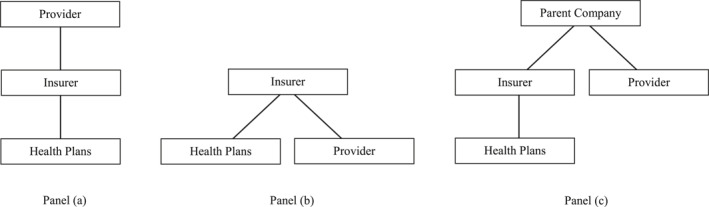
Simplified organizational chart for provider‐insurer integrated systems. Panel (a) illustrates a healthcare organization (referred to as “provider”) owning an insurer, panel (b) reflects an insurer owning a provider, panel (c) shows an insurer and a provider owned by the same parent company. Figure 1 shows health plans as products of insurers. An insurance company may operate in multiple insurance markets and regions, and can offer multiple products, a.k.a. health plans, in a specific insurance market or region.In practice, the ownership structures of provider‐insurer integrated systems are more complex than shown in Figure 1.

Prior empirical studies have identified provider‐insurer integrations using binary classifications with varying definitions (Allan Baumgarten, LLC 2017; Frakt et al. 2013; Howard et al. 2018; La Forgia et al. 2018; Meyers et al. 2020). The ownership structures in these definitions can be categorized into three simplified forms (Figure 1, panels (a)‐(c)): (a) a provider owns an insurer (Allan Baumgarten, LLC 2017), (b) an insurer owns a provider (Howard et al. 2018), and (c) an insurer and a provider are owned by the same parent company (Frakt et al. 2013; La Forgia et al. 2018; Meyers et al. 2020). Some studies have focused on one form, while others included all three. This article includes all three, with ownership terms referring to either full or partial ownership (see illustrative examples in Supporting Information S1: Appendix Section 2).

While classifying integration status as “Yes” or “No” often simplifies analyses, it can obscure important features. For example, La Forgia et al. (2018) were cautious in classifying insurers integrated solely with primary care providers as “integrated”. Whereas, primary care is the first point of care for most individuals and associated with enhanced healthcare access, better health outcomes, and reduced high‐cost care use (Shi 2012). Meyers et al. (2020) considered hospitals offering Medicare Advantage plans to be “integrated”. However, hospitals that offer only commercial health plans may still accept Medicare Advantage beneficiaries.

## 4 Variations in Provider‐Insurer Integrations

### 4.1 Care Delivery: With Which Providers is an Insurer integrated?

Integrated systems may own various providers that offer different levels of care. Some integrated systems own hospitals, while others only own primary care providers (La Forgia et al. 2018; Meyers et al. 2020). According to the Agency for Healthcare Research and Quality (AHRQ) Compendium data, approximately 38%–43% of hospitals belonged to a health system that offered insurance products from 2016 to 2022. Only 9%–12% of medical group practices providing primary care services were linked to health systems that offered insurance products. Although 80%–86% of medical group practices providing primary care services were not linked to health systems, some of them may be integrated with insurance companies, such as Optum Care, which is owned by UnitedHealth Group (Supporting Information S1: Appendix Table 1) (AHRQ 2024). The proportion might be low, as under 5% of physicians who completed the American Medical Association (AMA) Physician Practice Benchmark Surveys reported working in practices wholly owned by Health Maintenance Organizations or Managed Care Organizations from 2012 to 2022 (Kane 2023).

The levels of care owned by an integrated system can also vary across regions. For example, Anthem Inc. (now Elevance Health Inc.) had two subsidiaries delivering primary care and specialty care, and one subsidiary providing palliative care in March 2022. However, in Wisconsin, only the palliative care subsidiary operated (Supporting Information S1: Appendix Section 3).

The levels of care owned by an integrated system in a region can affect its considerations for selecting in‐network providers, including the reimbursement models specified in contracts (Ghili 2022). Integrated systems owning hospitals may have a stronger negotiating position when dealing with other hospitals in the same region compared to those that solely own primary care practices (Ghili 2022). Moreover, coordination among in‐network providers may vary depending on whether they belong to the same system. Providers belonging to the same system may have closer professional relationships (Agha et al. 2023). Information exchange can be easier and more comprehensive within one system due to streamlined coordination technology and fewer filtering entities (Agha et al. 2023; Hart and Holmstrom 2010; Williamson 1971). These can lead to variation in care utilization and care quality (Agha et al. 2022; Agha et al. 2023; Frakt et al. 2013; Meyers et al. 2020).

### 4.2 Insurance Markets: In Which Insurance Markets Does an Integrated System Compete?

When offering insurance products, integrated systems can choose in which insurance markets to compete. For example, among health systems offering insurance products, approximately 61%–69% offered Medicare Advantage plans from 2018 to 2022, while only around 41%–48% offered Medicaid managed care products (Supporting Information S1: Appendix Table 2).

Healthcare provider organizations and health insurers compete in multiple output and factor markets, including the healthcare workforce, health insurance products, and healthcare services. Policy concerns about vertical integration have often focused on patient care markets, particularly the effects of integration on the price and quality of care (Khullar et al. 2024; Rooke‐Ley et al. 2024). Therefore, this article focuses on patient care services.

Both the pricing and features of patient care services vary depending on the populations served, which are segmented by insurance product markets (e.g., Medicare, Medicaid, group, and individual markets). The pricing—including reimbursement mechanisms and fee schedules—and characteristics of patient needs are likely to be intertwined with integrated systems' competitive and care management strategies (Eggleston et al. 2004). For instance, Medicaid serves primarily the low‐income population, while Medicare covers elderly and disabled individuals. The rate of emergency department visits among Medicaid beneficiaries is nearly twice that of Medicare beneficiaries (Cairns et al. 2023). A navigation program for Medicaid beneficiaries might focus on reducing repeat ED visits, whereas a Medicare navigation program might focus on cancer patients (Bakshi et al. 2022; Rocque et al. 2017).

An integrated system's involvement in one or multiple insurance markets can also vary by region within the system. Additionally, healthcare providers may sometimes experiment with entering the insurance market by offering self‐funded health plans to their employees (Steele and Dafny 2016). Therefore, recognizing the types of insurance products an integrated system offers can help in understanding the systems' behavior.

### 4.3 Organizational Management: Is the Provider or the Insurer Dominant?

Including both a provider and an insurer under one umbrella often means one is dominant, typically the ultimate owner. The dominant party has more opportunities to shape the long‐term goals and competing strategies of the system. Sometimes, determining the dominance relationship can be challenging for outsiders, but clues may lie in an integrated system's revenue composition and board members.

When the provider dominates, the role of the integrated system's insurance products may be to support the provider's reforms, as with Geisinger Health System before it joined Risant Health (Supporting Information S1: Appendix Section 4). In these scenarios, the integrated system may have stronger incentives to steer patients from unintegrated providers toward its own provider (Cuesta et al. 2024). When the insurer dominates, the goal of owning providers can be to pay for outcomes rather than procedures, like UnitedHealth Group (UHG) (Supporting Information S1: Appendix Section 5). In such cases, the integrated system might have stronger incentives to lower the prices paid to unintegrated providers (Ghili 2022). The competing strategy may influence the net effect of the trade‐offs between the associated efficiency gains and anti‐competitive consequences.

Moreover, an integrated system with insurer dominance might be less inclined to own hospitals, as hospitals may care more about filling beds—an expensive way to provide care (Mendelson 2018). Providers that own hospitals may prefer to establish their own health plans to gain market strength and more control over premium revenue, rather than becoming insurer subsidiaries (Allan Baumgarten, LLC 2017).

### 4.4 The Dynamics of Provider‐Insurer Integrated Systems

The structure of an integrated system may continue evolving, and acquisitions and mergers may happen between distinct integrated systems. For example, Optum Health is the business line of UHG that supports and delivers care. From 2011 to 2022, its revenue from external customers increased from 2% to 9% of UHG's total revenue. Meanwhile, beneficiaries of UHG's insurance products surged from 31.7 million to 42 million (Supporting Information S1: Appendix Section 5). Risant Health, created by Kaiser Foundation Hospitals, acquired the Geisinger Health System in March 2024, and Cone Health in December 2024 (Cone Health 2024). Risant Health's goal is to find five to seven of these leading community‐based health systems over the next five to six years (Kaiser Permanente 2024; Raths 2025). These dynamics within and between integrated systems have the potential to influence healthcare organizations and insurance markets nation‐wide. However, a binary classification of provider‐insurer integration would not be able to reflect them.

Moreover, the number of beneficiaries enrolled in integrated health plans and receiving care from providers owned by the same integrated system (receiving fully integrated care) may reflect these dynamics. Quartz, a brand of health plans that has become well‐known since 2019 in Iowa, Illinois, Minnesota, and Wisconsin (Quartz 2024), was formed through a series of acquisitions from 2016 to 2017, involving three integrated systems. In 2015, within each of the three systems, a health system owned an insurance corporation. As a result of the acquisitions, the three insurance corporations became co‐owned by the three health systems, while the health systems themselves remained independent of one another (Supporting Information S1: Appendix Section 6). The group of beneficiaries receiving fully integrated care would become larger in this case, because enrollees covered by health plans from one of the three insurance corporations would receive fully integrated care if treated by any of the three health systems. Prior to the acquisitions, they would receive fully integrated care only if treated by the health system that owned their health plan.

## 5 Potential Applications

Based on our framework and taxonomy, we suggest several applications for empirical research. First, identify integrated systems based on research questions. For example, when potentially preventable hospitalizations are the concern, classifying insurer–primary care provider integrations into the *integrated* group might be beneficial. Although a binary classification can be used, sensitivity analyses could be informative when changing the definition of the *integrated* group to insurer–hospital integrations. Second, collect information on all insurance products offered by an integrated system, even if the research question focuses only on one insurance market. Competitive and care management strategies can extend beyond a single market. When a health system offers Medicare Advantage plans rather than Medicaid HMOs, a study focusing on the Medicaid market may need to consider whether to classify this health system as *integrated*. However, it's worth noting that patients of providers offering only self‐funded health plans may experience very limited impacts from this VI. Third, distinguish between beneficiaries of an integrated insurer receiving care from providers owned by the same integrated system and those receiving care from providers owned by a different integrated system. Instead of using a binary classification, grouping beneficiaries into multiple categories based on the degree of provider–insurer integrations they experienced may be useful in fulfilling this goal. This can help examine integrated system dynamics, as mentioned earlier.

The 2023 Merger Guidelines issued by the U.S. Department of Justice and the Federal Trade Commission convey an impression that they believe vertical mergers are more often harmful than beneficial (Carlton 2024). There might be closer scrutiny and more challenges to provider–insurer integrations in the future. However, each case has a uniquely complicated set of effects that may balance out to some extent. The framework suggested here provides insights into variations in provider‐insurer integrations and offers a reference for more comprehensive analysis when evaluating the relevant cases. It encompasses four aspects—care delivery, insurance markets, organizational management, and integrated system dynamics—crucial for studying how and why provider‐insurer integrations impact health outcomes, care utilization, and their conflicting values of coordination and competition.

## Conflicts of Interest

The authors declare no conflicts of interest.

## Supporting information

Supporting Information S1

## Data Availability

The data that support the findings of this study are openly available in the Compendium of U.S. Health Systems at https://www.ahrq.gov/chsp/data‐resources/compendium.html, and in the Doctors and Clinicians national downloadable files at https://data.cms.gov/provider‐data/archived‐data/doctors‐clinicians.
