# Supplementary material for: Classifying the Integration of Healthcare Providers and Insurers
Source: Health Econ. 2025 Jul 22;34(11):1971–6. doi: 10.1002/hec.70019 (PMC12496020; doi:10.1002/hec.70019)
Supplement: Supplementary file 1 — Supporting Information S1 [file HEC-34-1971-s001.docx]

## **Appendix**

### Brief

Combinations of prepayment for health care and care delivery within a single organization (provider–insurer integrations) have existed in the U.S. since as early as the nineteenth century, although the early arrangements may not have been designed to offer comprehensive care.

San Francisco’s French Hospital, built in 1852 by the French Mutual Benevolent Society, provided prepaid care for $1 a month to thousands of the city’s French immigrants (Kaplan, 2022; Stewart, 2004). The forerunner of the far-reaching Blue Cross hospital insurance was also a prepayment plan for twenty-one days of hospitalization at Baylor Hospital. It was designed in 1929 by the vice president of Baylor University, who oversaw the hospital (Minor, 1976).

A specific arrangement, known as Prepaid Group Practice (PGP), emerged when the Ross-Loos Clinic was established in 1929. PGPs combine prepayment for health care with comprehensive health care delivery. They began expanding in the 1930s and 1940s, an era when they were fighting for legitimacy and when the origins of Kaiser Permanente appeared (Christianson & Avery, 2004; Enthoven & Tollen, 2004; Stewart, 2004).

After Medicare and Medicaid were enacted in 1965 (National Archives, 2022), health policies shifted their focus towards controlling costs. The passage of the Health Maintenance Organization (HMO) Act and the envisioning of the “managed competition” concept by Alain Enthoven encouraged the formation of provider-insurer integration from the 1980s to the 1990s (Christianson & Avery, 2004; Allan Baumgarten, LLC, 2017; Enthoven, 1993). The rising concerns about care quality led to the “managed care backlash” around 2000. It posed challenges to provider-insurer integrations (Christianson & Avery, 2004; Miller, 2006). The most recent surge of provider-insurer integrations is promoted by the paying-for-value under the Affordable Care Act(ACA) (Allan Baumgarten, LLC, 2017).

### Illustrative Examples of U.S. Provider-Insurer Integrations

This paper considers both full and partial vertical integration of insurance company and healthcare provider through ownership to be provider-insurer integrations. To provide a better sense, this section discusses some well-known arrangements in U.S. healthcare system.

Firstly, this paper follows the definition of health insurance product provided by 45 CFR §144.103 (10–1–24 Edition)—"a discrete package of health insurance coverage benefits that are offered using a particular product network type (such as health maintenance organization (HMO), preferred provider organization (PPO), exclusive provider organization, point of service, or indemnity) within a service area."(*45 CFR § 144.103*, 2024) An insurance company may issue insurance products under various business lines, including individual, group, Medicare advantage as well as Medicaid. Therefore, whether the insurance products are Medicare Advantage plans, managed care organizations for Medicaid, individual or group insurance market HMOs or PPOs, if they are offered by an insurance company that is vertically integrated with a healthcare organization, these insurance products are considered to belong to a provider-insurer integrated system.

Secondly, both private entities and public agencies can form provider–insurer integrations. For example, Kaiser Permanente is considered a provider–insurer integrated system because it comprises Kaiser Foundation Health Plan, Inc. (an insurance company), Kaiser Foundation Hospitals, and the Permanente Medical Groups (healthcare organizations) (Kaiser Permanente, 2025). Additionally, this paper considers the U.S. Department of Veterans Affairs (VA) a provider–insurer integrated system. The VA provides health coverage for eligible members (insurance products) and owns medical centers and clinics (healthcare organizations) (U.S. Department of Veterans Affairs, 2024, 2025).

Furthermore, local governments that own public hospitals and issue their own health insurance products are also considered provider–insurer integrated systems. For instance, Hennepin Healthcare System, Inc. (a healthcare organization) is a component unit of Hennepin County in Minnesota (Hennepin Healthcare, 2024). Hennepin County also owns a health maintenance organization, Hennepin Health (Hennepin Health, 2025). Therefore, this paper considers Hennepin Healthcare System, Inc. and Hennepin Health to belong to a provider-insurer integrated system. However, if Hennepin Healthcare System, Inc. or Hennepin Health were owned by the state or another county rather than both being owned by Hennepin County, this paper would not consider them to belong to an integrated system. We would also like to note that this paper does not consider a public hospital to belong to an integrated system solely because it treats a large proportion of indigent patients.

### Anthem

Anthem, Inc. changed its name to Elevance Health, Inc. in 2022 and reorganized its business lines under three brands: Anthem Blue Cross/Anthem Blue Cross and Blue Shield, Wellpoint, and Carelon (*Elevance Health, Inc. Form 10-K 2022.*, 2023).

Anthem, Inc. had many subsidiaries, among which we identified three that were operating healthcare services as of March 2022: CareMore, HealthSun, and Aspire Health (“Anthem Affiliated/Specialty Companies,” 2022; *Anthem Inc. Form10-K 2021.*, 2022). On January 1, 2024, CareMore and Aspire Health became Carelon Health (Carelon Health, 2024a).

CareMore stated that its Care Centers are one-stop outpatient facilities (“About CareMore,” 2023). Based on information from CareMore's "Who We Are" webpage, CareMore operated in nine states and D.C. as of March 2022 (CareMore, 2021). (As of August 2024, Carelon Health’s advanced primary care still serves nine states and D.C.(Carelon Health, 2024b))

HealthSun was acquired by Anthem, Inc. in 2017. At that time, HealthSun owned 19 Pasteur and WellMax primary care and specialty centers in Florida (Business Wire, 2017a, 2017b). As of August 2024, HealthSun continues to operate WellMax Medical Center and Pasteur Medical Center in Florida (Pasteur Medical Center, 2023; WellMax Medical Centers, 2023).

Anthem, Inc. completed the acquisition of Aspire Health on June 18, 2018 (“Anthem, Inc. Completes Acquisition of Aspire Health,” 2018). As of March 2023, we found that Aspire Health operates an office in Milwaukee, Wisconsin (“Aspire Offices in Wisconsin,” 2023). Aspire Health treats patients with complex conditions or suffering from serious illnesses such as cancer, congestive heart failure (CHF), chronic obstructive pulmonary disease (COPD), kidney failure, liver failure, advanced dementia, and amyotrophic lateral sclerosis (ALS) (“Aspire Health-Frequently Asked Questions,” 2022).

### Geisinger

Geisinger Health System is a prominent integrated system established in 1915 in Pennsylvania (Raths, 2025; Geisinger, 2023). Its former President and CEO, Glenn D. Steele, stated, “If I could do it (care redesign) without necessarily having to set up my own payer…that would be my preference.”(Steele & Dafny, 2016)

### UnitedHealth Group (UHG)

UnitedHealth Group (UHG) owns both an insurance arm, UnitedHealthcare, and an arm that delivers care, Optum. Its insurance arm generated over 77% of UHG’s revenue in 2022 (UnitedHealth Group, 2023).

In 2011, UHG announced Optum as the complementary arm to its insurance business, UnitedHealthcare, and reorganized three business lines under Optum (FIERCE Healthcare, 2011). Optum Health is the one that supports and delivers care. It serves both external and internal customers (UnitedHealth Group, 2023). An article introducing UnitedHealthcare states, “The capabilities of both companies (UnitedHealthcare and Optum) help power better health outcomes, lower costs, and simpler experiences.”(UnitedHealthcare, 2023)

From 2011 to 2022, Optum Health’s revenue from external customers increased from 2% to 9% of UHG’s total revenue. Optum Health’s transactions with internal businesses also increased, although the growth rate (approximately 22%) was not as pronounced as the revenue increase from external customers (exceeded 26%). Concurrently, UnitedHealthcare’s U.S. enrollees surged from 31.7 million to 42 million from 2011 to 2022.

The percentage of UnitedHealth Group’s revenue from Optum Health and the growth rate of Optum Health transactions with external and internal customers are calculated based on information from “Note 13” to the “Consolidated Financial Statements” of UnitedHealth Group’s 2011 Annual Report(UnitedHealth Group, 2012) and “Note 14” to the “Consolidated Financial Statements” of UnitedHealth Group’s 2022 Annual Report(UnitedHealth Group, 2023)—Segment Financial Information. Please note that the intersegment transactions of UnitedHealth Group were recorded at management’s estimate of fair value and are not counted toward UnitedHealth Group’s total revenue in its consolidated financial statements.

As of December 31, 2011, UnitedHealthcare Employer & Individual offered a comprehensive array of consumer-oriented plans and services for nearly 26 million people in the U.S.; UnitedHealthcare Medicare & Retirement served about 2.2 million people through its Medicare Advantage products; UnitedHealthcare Community & State participated in programs in 23 states and the District of Columbia and served 3.5 million people (UnitedHealth Group, 2012).

As of December 31, 2022, UnitedHealthcare Employer & Individual provided access to medical services for 26.7 million people in the U.S.; UnitedHealthcare Medicare & Retirement served 7.1 million people through its Medicare Advantage products; UnitedHealthcare Community & State participated in programs in 35 states and the District of Columbia and served 8.2 million people (UnitedHealth Group, 2023).

### Quartz

Gundersen Health Plan (GHP) was incorporated in Wisconsin on March 13, 1995, by Gundersen Clinic, Ltd. (the Clinic) and Gundersen Lutheran Medical Center, Inc. (the Hospital). On January 1, 2000, Gundersen Lutheran, Inc., an affiliation created by the Clinic and the Hospital in 1996, became the new parent of Gundersen Health Plan, replacing the Clinic and the Hospital. On March 26, 2013, Gundersen Lutheran, Inc., changed its legal name to Gundersen Health System (Wisconsin Office of the Commissioner of Insurance, 2019a).

Unity Health Plans Insurance Corporation is incorporated as HMO of Wisconsin on October 31, 1983. On November 1, 1994, United Wisconsin Services, Inc. acquired HMO of Wisconsin as well as the insurance business of U-Care HMO, Inc. And HMO of Wisconsin assumed both the benefit and provider contracts of U-Care HMO, Inc. On April 1, 1995, HMO of Wisconsin changed its name to Unity Health Plans Insurance Corporation. On January 1, 2005, Unity Health Plans Insurance Corporation was acquired by University Health Care, Inc. (UHC) as a wholly-owned subsidiary, where UW Health is the parent of UHC (UW Health, 2022; Wisconsin Office of the Commissioner of Insurance, 2019c).

Physicians Plus Insurance Corporation is a life, accident, and health (LAH) insurance company. The company was incorporated on July 17, 1986. On December 31, 2013, Meriter Health Services, Inc., purchased 100% of Physicians Plus Insurance Corporation stock. On February 1, 2014, Meriter Health Services, Inc., sold its shares to Iowa Health System d/b/a Unity Point Health (UPH), a provider of health-related services that operates in Iowa and Northern Illinois (Wisconsin Office of the Commissioner of Insurance, 2019b).

On April 21, 2016, acquisitions of Gundersen Health Plan by University Health Care (a subsidiary of UW Health) and Unity Health Plans by Gundersen Lutheran got approved (Quartz 2016 transaction). As a result, each of Gundersen Health Plan and Unity Health Plans became co-owned by Gundersen Health System and UW Health (UW Health, 2022; Wisconsin Office of the Commissioner of Insurance, 2016). On June 29, 2017, the application regarding the transaction to add Physician Plus Insurance Corporation into the structure built by the Quartz 2016 transaction got approved. Quartz Holding Company was created in the 2017 transaction. Consequently, Gundersen Health Plan, Unity Health Plans Corporation and Physician Plus Insurance Corporation were all co-owned by Gundersen Health System, UW Health, and UnityPoint Health, but these health systems remained independent of each other after the 2017 transaction (Wisconsin Office of the Commissioner of Insurance, 2021).

### Table 1

|  | 2016 | | 2018 | | 2020 | | 2021 | | 2022 | |
| --- | --- | --- | --- | --- | --- | --- | --- | --- | --- | --- |
|  | Num. | % | Num. | % | Num. | % | Num. | % | Num. | % |
| # of hospitals | 6,762 | 100.0% | 6,742 | 100.0% | 6,701 | 100.0% | 6,725 | 100.0% | 6,764 | 100.0% |
| not linked to health systems (HS) | 2,813 | 41.6% | 2,855 | 42.3% | 2,664 | 39.8% | 2,652 | 39.4% | 2,591 | 38.3% |
| linked to HS | 3,949 |  | 3,887 |  | 4,037 |  | 4,073 |  | 4,173 |  |
| Missing data on insurance products | 48 | 0.7% | 69 | 1.0% | 172 | 2.6% | 115 | 1.7% | 116 | 1.7% |
| # of HS didn't offer any insurance products | 998 | 14.8% | 1119 | 16.6% | 1340 | 20.0% | 1370 | 20.4% | 1419 | 21.0% |
| # of HS offered insurance products | 2,903 | 42.9% | 2,699 | 40.0% | 2,525 | 37.7% | 2,588 | 38.5% | 2,638 | 39.0% |
|  |  |  |  |  |  |  |  |  |  |  |
| # of medical groups | 41,153 | 100.0% | 39,103 | 100.0% | 37,109 | 100.0% | 36,202 | 100.0% | 35,448 | 100.0% |
| not linked to HS | 35,823 | 87.0% | 32,946 | 84.3% | 31,015 | 83.6% | 30,270 | 83.6% | 29,617 | 83.6% |
| linked to HS | 5,330 |  | 6,157 |  | 6,094 |  | 5,932 |  | 5,831 |  |
| Missing data on insurance products | 81 | 0.2% | 162 | 0.4% | 307 | 0.8% | 210 | 0.6% | 202 | 0.6% |
| # of HS didn't offer any insurance products | 1765 | 4.3% | 2153 | 5.5% | 2164 | 5.8% | 2162 | 6.0% | 2101 | 5.9% |
| # of HS offered insurance products | 3,484 | 8.5% | 3,842 | 9.8% | 3,623 | 9.8% | 3,560 | 9.8% | 3,528 | 10.0% |
|  |  |  |  |  |  |  |  |  |  |  |
| # of medical groups providing primary care | 21,955 | 100.0% | 21,446 | 100.0% | 22,447 | 100.0% | 22,097 | 100.0% | 21,632 | 100.0% |
| not linked to HS | 18,935 | 86.2% | 17,297 | 80.7% | 18,107 | 80.7% | 17,783 | 80.5% | 17,400 | 80.4% |
| linked to HS | 3,020 |  | 4,149 |  | 4,340 |  | 4,314 |  | 4,232 |  |
| Missing data on insurance products | 38 | 0.2% | 112 | 0.5% | 218 | 1.0% | 155 | 0.7% | 152 | 0.7% |
| # of HS didn't offer any insurance products | 1046 | 4.8% | 1462 | 6.8% | 1571 | 7.0% | 1599 | 7.2% | 1562 | 7.2% |
| # of HS offered insurance products | 1,936 | 8.8% | 2,575 | 12.0% | 2,551 | 11.4% | 2,560 | 11.6% | 2,518 | 11.6% |

Note: Appendix Table 1 was generated based on data from the Agency for Healthcare Research and Quality (AHRQ) Compendium (2016–2022) and the Doctors and Clinicians national downloadable files (AHRQ, 2024; CMS.gov, 2025). Medical groups providing primary care were identified by combining these two datasets. This article considers medical groups that include at least one primary care provider, either a physician or non-physician practitioner, providing primary care services. Primary care providers are those whose modest (most common) specialty listed at their affiliated practice is one of the following: Family Practice, General Practice, Geriatric Medicine, Internal Medicine, Nurse Practitioner, Pediatric Medicine, Physician Assistant, Preventive Medicine, Certified Nurse Midwife, or Obstetrics/Gynecology. About 15% to 20% of medical group practices in the AHRQ Compendium can’t be matched with those in the National Physician Downloadable files. Thus, it is not clear whether they provided primary care.

### Table 2

|  | 2016 | | 2018 | | 2020 | | 2021 | | 2022 | |
| --- | --- | --- | --- | --- | --- | --- | --- | --- | --- | --- |
|  | Num. | % | Num. | % | Num. | % | Num. | % | Num. | % |
| Total # of health systems (HS) | 626 |  | 637 |  | 629 |  | 635 |  | 640 |  |
| Missing data on insurance products | 25 |  | 40 |  | 86 |  | 60 |  | 61 |  |
| # of HS didn't offer any insurance products | 357 |  | 383 |  | 349 |  | 368 |  | 370 |  |
| # of HS offered insurance products | 244 | 100.0% | 214 | 100.0% | 194 | 100.0% | 207 | 100.0% | 209 | 100.0% |
| *Medicare Advantage (MA)* | *74* | *30.3%* | *130* | *60.7%* | *133* | *68.6%* | *137* | *66.2%* | *141* | *67.5%* |
| *Medicaid* | *n/a* |  | *102* | *47.7%* | *78* | *40.2%* | *85* | *41.1%* | *85* | *40.7%* |
| *Individual Marketplace* | *n/a* |  | *82* | *38.3%* | *66* | *34.0%* | *79* | *38.2%* | *80* | *38.3%* |
| *MA&Medicaid* | *n/a* |  | *72* | *33.6%* | *59* | *30.4%* | *66* | *31.9%* | *67* | *32.1%* |
| *MA&Individual Marketplace* | *n/a* |  | *63* | *29.4%* | *59* | *30.4%* | *69* | *33.3%* | *72* | *34.4%* |
| *Medicaid & Individual Marketplace* | *n/a* |  | *58* | *27.1%* | *46* | *23.7%* | *56* | *27.1%* | *56* | *26.8%* |
| *MA&Medicaid & Individual Marketplace* | *n/a* |  | *50* | *23.4%* | *43* | *22.2%* | *53* | *25.6%* | *54* | *25.8%* |

Note: Appendix Table 2 was generated based on data from the Agency for Healthcare Research and Quality (AHRQ) Compendium (2016–2022)(AHRQ, 2024). From 2018 to 2022, the identification of offering Medicare Advantage (MA) plans was based on both the American Hospital Association (AHA) survey and the MA Plan Directory. However, the AHA survey used in the 2016 AHRQ Compendium data file did not identify the type of insurance products. Thus, only the MA Plan Directory was used to identify whether a health system offered an MA plan that year.

### Appendix References:

*45 CFR § 144.103*. (2024). Retrieved from https://www.govinfo.gov/content/pkg/CFR-2024-title45-vol2/pdf/CFR-2024-title45-vol2-sec144-103.pdf

About CareMore. (2023). Retrieved April 7, 2023, from CareMore Health website: https://www.caremore.com/About-Us/About-CareMore-Health.aspx#

AHRQ. (2024, November). Compendium of U.S. Health Systems. Retrieved February 21, 2024, from Agency for Healthcare Research and Quality website: https://www.ahrq.gov/chsp/data-resources/compendium.html

Allan Baumgarten, LLC. (2017). *Analysis of Integrated Delivery Systems and New Provider- Sponsored Health Plans*. 30.

Anthem Affiliated/Specialty Companies. (2022). Retrieved March 26, 2022, from https://www.antheminc.com/Companies/AffiliatedSpecialtyCompanies/index.htm

Anthem, Inc. Completes Acquisition of Aspire Health. (2018, June 18). Retrieved April 7, 2023, from https://www.elevancehealth.com/newsroom/anthem-inc-completes-acquisition-of-aspire-health

*Anthem Inc. Form10-K 2021.* (2022). Retrieved from https://ir.antheminc.com/financial-information/sec-filings

Aspire Health-Frequently Asked Questions. (2022). Retrieved March 26, 2022, from https://aspirehealthcare.com/frequently-asked-questions/

Aspire Offices in Wisconsin. (2023). Retrieved March 18, 2023, from https://aspirehealthcare.com/state/WI/

Business Wire. (2017a, September 20). Anthem to Acquire HealthSun. Retrieved April 7, 2023, from https://www.businesswire.com/news/home/20170920005561/en/Anthem-to-Acquire-HealthSun

Business Wire. (2017b, December 21). Anthem Completes Acquisition of HealthSun. Retrieved from https://www.businesswire.com/news/home/20171221005587/en/Anthem-Inc.-Completes-Acquisition-of-HealthSun

Carelon Health. (2024a). About Us. Retrieved February 18, 2024, from https://www.carelonhealth.com/about-us

Carelon Health. (2024b). States we serve. Retrieved February 18, 2024, from https://www.carelonhealth.com/advanced-primary-care/states

CareMore. (2021, September 20). Who We Are. Retrieved March 26, 2022, from CareMore Health website: https://www.caremore.com/Who-We-Are.aspx

Christianson, J. B., & Avery, G. (2004). Chapter 4 Prepaid Group Practice and Health Care Policy. In A. C. Enthoven & L. A. Tollen (Eds.), *Toward a 21st Century Health System: The Contributions and Promise of Prepaid Group Practice* (1st ed., pp. 61–84). John Wiley & Sons.

CMS.gov. (2025). *The Doctors and Clinicians national downloadable file* [Data set]. Retrieved from https://data.cms.gov/provider-data/archived-data/doctors-clinicians

*Elevance Health, Inc. Form 10-K 2022.* (2023). Retrieved from https://ir.antheminc.com/financial-information/sec-filings

Enthoven, A. C. (1993). The History and Principles of Managed Competition. *Health Affairs*, *12*(suppl 1), 24–48. https://doi.org/10.1377/hlthaff.12.Suppl_1.24

Enthoven, A. C., & Tollen, L. A. (2004). Preface. In A. C. Enthoven & L. A. Tollen (Eds.), *Toward a 21st Century Health System: The Contributions and Promise of Prepaid Group Practice* (1st ed., pp. xxvii–xivii). John Wiley & Sons.

FIERCE Healthcare. (2011, April 11). *UnitedHealth Group Announces “Optum” Master Brand for its Health Services Businesses*. Retrieved from https://www.fiercehealthcare.com/healthcare/unitedhealth-group-announces-optum-master-brand-for-its-health-services-businesses

Geisinger. (2023). Geisinger-Who We Are. Retrieved from https://www.geisinger.org/about-geisinger/who-we-are

Hennepin Health. (2025). Who we are_Hennepin Health. Retrieved March 21, 2025, from https://www.hennepinhealth.org/about-us/about

Hennepin Healthcare. (2024). *Hennepin Healthcare System, Inc. 2023 Financial Report*. Retrieved from https://www.hennepinhealthcare.org/wp-content/uploads/2024/06/Hennepin-Healthcare-System_23-FS_Final.pdf

Kaiser Permanente. (2025). Our impact_Kaiser Permanente. Retrieved March 21, 2025, from https://about.kaiserpermanente.org/commitments-and-impact/public-policy/our-impact

Kaplan, F. (2022, July 1). The French Hospital, San Francisco. Retrieved from https://californiahistoricalsociety.org/blog/the-french-hospital-san-francisco/

Miller, N. H. (2006). Insurer-Provider Integration, Credible Commitment, and Managed-Care Backlash. *Journal of Health Economics*, *25*(5), 861–876. https://doi.org/10.1016/j.jhealeco.2005.12.007

Minor, D. (1976). Justin Ford Kimball: Pioneer of Blue Cross Hospital Insurance and Education Leader. Retrieved from https://www.tshaonline.org/handbook/entries/kimball-justin-ford

National Archives. (2022). *Medicare and Medicaid Act (1965)*. Retrieved from https://catalog.archives.gov/id/299908

Pasteur Medical Center. (2023). Home. Retrieved April 7, 2023, from https://pasteurmedical.com/

Raths, D. (2025, May 12). Risant Health’s 4 Criteria as It Looks to Grow Beyond Geisinger, Cone Health. *Healthcare Innovation*. Retrieved from https://www.hcinnovationgroup.com/policy-value-based-care/risk-based-contracting/article/55289727/risant-healths-4-criteria-as-it-looks-to-grow-beyond-geisinger-cone-health

Steele, G. D., & Dafny, L. (2016). When Do Provider-Sponsored Health Plans Make Sense? Part 1. *NEJM Catalyst*. Retrieved from https://catalyst.nejm.org/doi/full/10.1056/CAT.16.0881

Stewart, J. A. (2004). Appendix: The Origins of Prepaid Group Practice in The United States. In A. C. Enthoven & L. A. Tollen (Eds.), *Toward a 21st Century Health System: The Contributions and Promise of Prepaid Group Practice* (1st ed., pp. 265–274). John Wiley & Sons.

UnitedHealth Group. (2012). *UnitedHealth Group Incorporated, Form 10-K 2011*. Retrieved from https://www.sec.gov/Archives/edgar/data/0000731766/000073176612000009/unh2011123110k.htm

UnitedHealth Group. (2023). *UnitedHealth Group Incorporated, Form 10-K 2022*. Retrieved from https://www.sec.gov/Archives/edgar/data/731766/000073176623000008/unh-20221231.htm#i6b660947fab7488cb4d33baca2cb3a37_259

UnitedHealthcare. (2023, November). *Combining the Capabilities of UnitedHealthcare and Optum for Greater Health Plan Value*. Retrieved from https://www.uhc.com/content/dam/uhcdotcom/en/e-i-articles/pdfs/tri3-enterprise-enabled-caps-article.pdf

U.S. Department of Veterans Affairs. (2024, August 24). About VA health benefits. Retrieved March 19, 2025, from https://www.va.gov/health-care/about-va-health-benefits/

U.S. Department of Veterans Affairs. (2025, January 20). Veterans Health Administration. Retrieved March 19, 2025, from https://www.va.gov/health/aboutvha.asp

UW Health. (2022). UW Health-University Health Care Inc. Retrieved May 4, 2022, from https://www.uwhealth.org/about-us/university-health-care#6pcq1jIMbzpu7LalVIYkv4

WellMax Medical Centers. (2023). Home. Retrieved April 7, 2023, from https://wellmaxmedicalcenters.com/

Wisconsin Office of the Commissioner of Insurance. (2016). *Proposed Acquisitions of Gundersen Health Plan by University Health Care and Unity Health Plans by Gundersen Lutheran (Approved on April 21, 2016)*. Retrieved from https://oci.wi.gov/Pages/Companies/GundersenUnityAcquisition.aspx

Wisconsin Office of the Commissioner of Insurance. (2019a). *Report of the Examination of Gundersen Health Plan, Inc. As of December 31, 2017*. Retrieved from https://oci.wi.gov/Documents/Companies/FinGundHealth.pdf

Wisconsin Office of the Commissioner of Insurance. (2019b). *Report of the Examination of Physicians Plus Insurance Corporation, as of December 31, 2017*. Retrieved from https://oci.wi.gov/Documents/Companies/FinPhyPlus.pdf

Wisconsin Office of the Commissioner of Insurance. (2019c). *Report of the Examination of Unity Health Plans Insurance Corporation, as of December 31, 2017*. Office of the Commissioner of Insurance, State of Wisconsin. Retrieved from Office of the Commissioner of Insurance, State of Wisconsin website: https://oci.wi.gov/Documents/Companies/FinUnity.pdf

Wisconsin Office of the Commissioner of Insurance. (2021). *Acquisitions of Gundersen Health Plan, Inc., and Unity Health Plans Insurance Corporation and Physicians Plus Insurance Corporation (Approved on June 29, 2017)*. Retrieved from https://oci.wi.gov/Pages/Companies/GundersenPhysiciansPlusAcquisitions.aspx
